# Dengue and COVID-19 Co-Circulation in the Peruvian Amazon: A Population-Based Study

**DOI:** 10.4269/ajtmh.22-0539

**Published:** 2023-04-24

**Authors:** Maria J. Pons, Ana Mayanga-Herrera, Gabriela M. Ulloa, Barbara Ymaña, Sabrina Medina, Freddy Alava, Carlos Álvarez-Antonio, Graciela Meza-Sánchez, Carlos Calampa, Wilma Casanova, Cristiam Carey, Hugo Rodríguez-Ferrucci, Amy C. Morrison, Antonio M. Quispe

**Affiliations:** ^1^ Grupo de Enfermedades Infecciosas Re-emergentes, Universidad Científica del Sur, Lima, Peru;; ^2^ Grupo Cultivo Celular e Immunología, Universidad Cientìfica del Sur, Lima, Peru;; ^3^ Dirección Regional de Salud de Loreto, Loreto, Peru;; ^4^ Universidad Nacional de la Amazonía Peruana, Loreto, Peru;; ^5^ Department of Pathology, Microbiology, and Immunology, School of Veterinary Medicine, University of California, Davis, California;; ^6^ Dirección de Investigación, Universidad Continental, Huancayo, Peru

## Abstract

The COVID-19 pandemic affected the main Amazon cities dramatically, with Iquitos City reporting the highest seroprevalence of anti-SARS-CoV-2 antibodies during the first COVID-19 wave worldwide. This phenomenon raised many questions about the possibility of a co-circulation of dengue and COVID-19 and its consequences. We carried out a population-based cohort study in Iquitos, Peru. We obtained a venous blood sample from a subset of 326 adults from the Iquitos COVID-19 cohort (August 13–18, 2020) to estimate the seroprevalence of anti-dengue virus (DENV) and anti-SARS-CoV-2 antibodies. We tested each serum sample for anti-DENV IgG (serotypes 1, 2, 3, and 4) and SARS-CoV-2 antibodies anti-spike IgG and IgM by ELISA. We estimated an anti-SARS-CoV-2 seroprevalence of 78.0% (95% CI, 73.0–82.0) and an anti-DENV seroprevalence of 88.0% (95% CI, 84.0–91.6), signifying a high seroprevalence of both diseases during the first wave of COVID-19 transmission in the city. The San Juan District had a lower anti-DENV antibody seroprevalence than the Belen District (prevalence ratio, 0.90; 95% CI, 0.82–0.98). However, we did not observe these differences in anti-SARS-CoV-2 antibody seroprevalence. Iquitos City presented one of the highest seroprevalence rates of anti-DENV and anti-SARS-CoV-2 antibodies worldwide, but with no correlation between their antibody levels.

## INTRODUCTION

As COVID-19 spread worldwide during 2020, several countries in tropical and subtropical regions reported simultaneous dengue epidemics.^1^^,^^2^ The convergence of these two diseases in the same space and time raised awareness about a potential syndemic, where the interaction between both conditions worsens the health outcomes.^3^ Currently, several cases of co-infections with COVID-19 and dengue virus (DENV) have been reported around the world,^4^^–^^7^ including in countries that share the Amazon Basin, such as Brazil,^8^ Colombia,^9^ and Peru.^10^ Both acute viral diseases—DENV and COVID-19—can be fatal for patients, and we know little about the impact of co-infection on diagnosis, prognosis, severity, and sequelae. In addition, there is concern about cross-reactivity in IgM/IgG detection tests for COVID and DENV.^11^ No significant efforts have been made to study SARS-CoV-2 and DENV co-infection.^12^ Before COVID-19, Peru was experiencing a DENV epidemic in the Peruvian Amazon basin and along the north coast of Peru.^13^ During 2020, Peru reported 56,394 cases of DENV and 88 deaths resulting from DENV, with most reported in Loreto (19.2% and 29.5%, respectively), a region with ∼1 million inhabitants that encompasses a third of Peru’s territory.^14^

The history of DENV transmission has been well characterized in Iquitos City since 1990, when a few years after the vector *Aedes aegypti* was reintroduced into the city after a nearly 30-year absence. Dengue virus transmission has been, and continues to be, reported annually, starting with dengue virus serotype 1 (DENV-1) in 1990,^15^^,^^16^ followed by the American genotype of serotype 2 in 1995,^17^ serotype 3 in 2001,^18^ and serotype 4 in 2008.^19^ At the end of 2010, the American-Asian genotype of serotype 2 caused a massive outbreak.^20^^,^^21^ It continued to circulate until the introduction of Zika virus^22^ in 2016, when almost no DENV transmission was observed until April 2017, after which DENV-2 began to be observed again. Ongoing cohort studies in the city have documented A high seroprevalence of anti-DENV antibodies that increase with age, reaching rates levels > 85% in adults.^23^^,^^24^

In this context, Peru experienced one of the most lethal first COVID-19 waves globally, with Iquitos as one of the first and hardest-hit cities in the country.^25^ In Iquitos, more than two thirds of total excess deaths occurred in a few weeks (April 19–May 18, 2020), primarily as a result of the collapse of the health-care system and the shortage of medical oxygen.^26^ After the first epidemic peak and under very challenging research conditions, we carried out a cohort study that estimated an anti-SARS-CoV-2 antibody seroprevalence of 70% (95% CI, 67–73) at baseline (July 13–18, 2020). This represented the highest reported anti-SARS-CoV-2 antibody seroprevalence worldwide at the time.^27^ To understand COVID-19 and DENV co-circulation further, we amended our cohort study to obtain venous blood samples from the adults in the cohort to conduct DENV serological assays. Herein, we report the results of this secondary study, the aim of which was to assess COVID-19 and dengue co-circulation by measuring the seroprevalence of anti-SARS-CoV-2 and anti-DENV IgG antibodies during August 2020.

## MATERIALS AND METHODS

### Study setting.

We conducted our study in Iquitos, a city of ∼467,000 inhabitants located in the department of Loreto in northeastern Peru (lat. 3.7°S, long. 73.2°W). We have described the study setting in detail previous.^27^ Briefly, the city has four districts: Iquitos (at the center), San Juan (south), Belen (east), and Punchana (north) (Supplemental Figure 1). Iquitos City is Loreto’s Department capital, and Loreto has the second-highest poverty level and extreme poverty rates (5.8%–7.9%) in Peru.^28^ Most of the population in the four districts that comprise Iquitos City are in urban neighborhoods, but there are several surrounding communities with populations of < 2,000 residents, which are classified as rural by the Peruvian government.^28^ At the end of 2019, cases of DENV-1 started to be detected; the number of cases increased each month until a quarantine was declared March 14, 2020. During this period, DENV-2 was present, but the proportion of DENV-1 cases had nearly replaced those of DENV-2 (A. C. M., unpublished data). Circulation of other flavivirus,^29^ as well as other arboviruses,^22^ have also been reported.

### Sample size.

Sample size was calculated to obtain the representative sample necessary to estimate an anti-SARS-CoV-2 antibody seroprevalence in the city of Iquitos of 18% with a precision of ±2.5% (50% relative error) at a 95% confidence level. Sampling effects were calculated to control for district differences in district population size, gender, and age group. We stratified the adult cohort into three age groups: 18 to 29 years, 30 to 59 years, and > 60 years. In anticipation of a 20% response rate, a 20% loss to follow-up, and 20% missing values, the minimum sample size increased to 692 subjects. We enrolled 726 participants at baseline to account for a 5% loss of information resulting from contingencies such as robbery, assault, or similar events. A total of 716 participants completed baseline sampling, including 429 adults. Of these adults, 326 (76.0%) completed the first follow-up and were tested for anti-SARS-CoV-2 and anti-DENV IgG antibodies. This sample size is large enough (power, > 80%) to estimate a seroprevalence as low as 10% with a precision of ±5% at a 95% CI.

### Study design.

**Figure 1.**
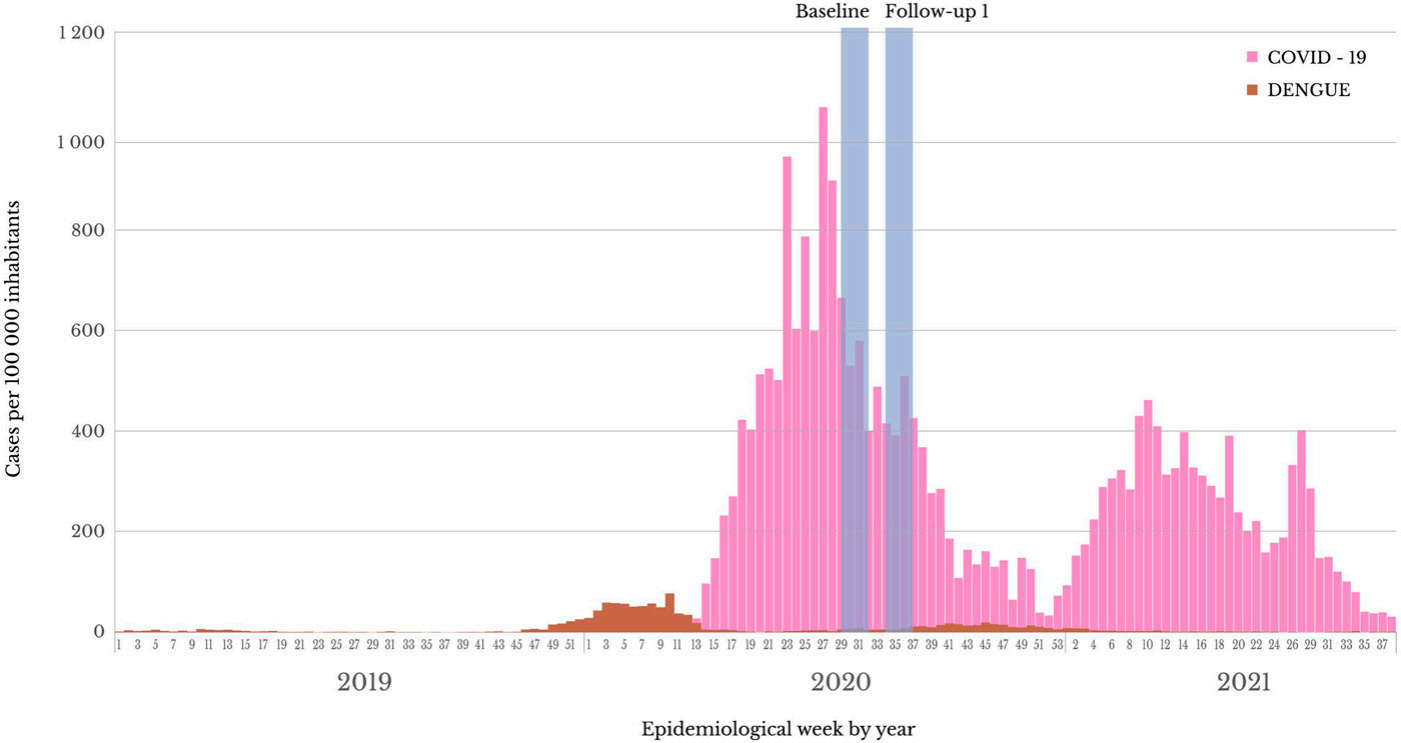
COVID-19 and dengue epidemics in Iquitos City from 2019 through 2021.

We leveraged a 3-month cohort study (July–September 2020) designed to estimate anti-SARS-CoV-2 antibody seroprevalence from a representative sample in the city. We have described the cohort study previously.^27^ Briefly, we obtained a representative sample of the Iquitos City population, which was distributed in 90,354 households located on 2,500 blocks in 40 sectors in 4 districts (Supplemental Figure 1). The sample was representative of each of the four districts, five age groups, and two genders of interest, so each district sample was weighted by gender and age group. In all cases, eligible participants were invited to participate, and all who gave their informed consent were surveyed. We attempted to screen each cohort participant during the first week of each month for 3 consecutive months (July, August, and September 2020). However, given the high anti-SARS-CoV-2 antibody seroprevalence observed in the cohort baseline (70%),^27^ we amended the study immediately to obtain a venous blood sample instead of a fingerstick during the first monthly follow-up, exclusively in our adult cohort. This sample was tested for SARS-CoV-2 by measuring IgM antibodies against SARS-CoV-2 spike protein S and for DENV antibodies by measuring IgG via ELISA (CTK Biotech, San Diego, CA). This sampling period corresponds to 3 months after the peak of the Iquitos COVID-19 epidemic curve (Figure 1).

### Inclusion and exclusion criteria.

All adults (≥ 18 years) with a baseline sample in the COVID-19 cohort study who were residents of Iquitos, Loreto, Peru, after the arrival of COVID-19 to Peru (March 6, 2020) were eligible for enrollment. Exclusion criteria included 1) institutionalized individuals at nursing homes, prisons, or boarding schools; 2) individuals with any contraindication for phlebotomy (cellulitis or abscess, venous fibrosis on palpation, presence of hematoma, vascular shunt or graft, or a vascular access device); and 3) health workers or individuals living with an active health worker.

### Procedures.

After providing consent for a larger blood sample, study personnel obtained a 4-mL blood sample by venipuncture and interviewed participants using standardized Ministry of Health epidemiological investigation forms. Next, blood samples were transported to the Dirección Regional de Salud Loreto laboratory in Iquitos for serum separation and were stored at –20°C until shipment in dry ice to the Universidad Científica del Sur laboratory in Lima, and subsequent testing for DENV and SARS-CoV-2 antibody determination using an ELISA.

### Laboratory analysis.

To detect anti-DENV IgG antibodies, we used a commercial ELISA kit that contained DENV-1, -2, -3, and -4 particles (RecombiLISA; CTK Biotech). Manufacturer specifications indicated a 96.4% sensitivity and a 98.1% specificity for the detection of acute dengue fever infection, including secondary infections. Unfortunately, anti-DENV IgM and non-structural protein 1 (NS1) testing were not possible because of the unavailability of kits in the country in the context of the pandemic. In addition, we tested the serum samples from the same individuals to detect anti-SARS-CoV-2 IgG with an ELISA that used the recombinant subdomain S1 of the spike protein as the antibody-capture antigen (EI 2606–9601 G; Euroimmun, Germany), with a 95.8% sensitivity and a 100.0% specificity, according to the manufacturer. Both anti-DENV and anti-SARS-CoV-2 IgG antibodies were evaluated by calculating a ratio of sample absorbance to calibrator absorbance (a ratio of > 1 was considered positive), indicating a recent infection, as defined by the manufacturer. Last, we tested the same serum samples to detect IgM anti-spike of SARS-CoV-2 with an ELISA that used the recombinant subdomain S1 of the spike protein as an antibody-capture antigen with an in-house protocol adapted by Stadlbauer et al.^30^ All samples were considered positive for IgG and IgM (DENV and SARS-CoV-2) if the absorbance units were greater than the mean of negative controls plus three times the SD of the controls. The absorbance values were read using a Biotek Synergy LX Multi-Mode Reader (Agilent) (Supplemental Table 1).

### Outcome definitions.

Two primary outcomes were considered: 1) presence of anti-DENV IgG, regardless of clinical signs and symptoms, as a proxy of a previous DENV infection; and 2) presence of IgG and IgM anti spike of SARS-CoV-2, regardless of clinical signs and symptoms, as a proxy of a COVID-19 previous infection.

### Clinical assessment.

After enrollment, we interviewed the study participants for COVID-19–like symptoms using the first standardized Ministry of Health epidemiological investigation form (March 2020). This form used the exact operational definition proposed by the CDC at the time. It included the following COVID-19–like symptoms: cough, fever (reported or measured), difficulty breathing, throat sore, nasal congestion, nausea or vomiting, diarrhea, and loss of smell or taste. In addition, we defined as asymptomatic those infection episodes without any of the COVID-19–like symptoms. Last, we described as symptomatic those infection episodes with at least one acute or worsening COVID-19–like symptom.

### Statistical analysis.

We performed a descriptive analysis summarizing participant demographics and clinical history, with absolute and relative frequencies if categorical, and mean and range if continuous. Then, we estimated the seroprevalence for each outcome using a two-step process. First, we estimate the seroprevalence by accounting for the survey sampling weights (which control for district population size, gender, and age group distribution) using STATA survey (svy) command. Second, we adjusted these estimates to account for the sensitivity (95.8% for SARS-CoV-2 and 96.4% sensitivity for DENV) and specificity (100% for SARS-CoV-2 and 98.1% for DENV) using the diagnostic (diagti) command. Third, we explored bivariate associations among the levels of the antibodies of interest by calculating Spearman’s rank correlation coefficients. Last, we explored associated factors to the two outcomes of interest, including the seroprevalence of anti-SARS-CoV-2 and DENV antibodies. In all cases, we estimated the prevalence ratio as the magnitude of association of interest using log-binomial regression models with robust variance and a CI of 95%. We analyzed all the data using the statistical package STATA^tm^ MP version 14.0 (StataCorp LP, College Station, TX).

## RESULTS

### Study population.

A total of 326 adults were included in the study. Samples from these participants were tested for anti-DENV and anti-SARS-CoV-2 antibodies, representing 80% of all adults who had provided baseline samples for the COVID-19 cohort study from Iquitos.^27^ We compared adults providing samples for DENV testing with all adults lost to follow-up between the baseline sample and the DENV sampling period. There were no significant differences between these two groups based on gender (*P* = 0.112), age (*P* = 0.991), or location (rural/urban) (*P* = 0.345).

### Population characteristics.

Mean participant age was 40.2 years (range, 19–88 years age), and all participants were asymptomatic. Most were women (54.6%), from the San Juan (38.7%) or Iquitos (32.8%) districts, and lived in urban areas (88.0%) (Table 1). Most patients denied any history of previously existing conditions (78.3%), whereas the following were reported: cardiovascular diseases (11.8%), diabetes (4.0%), kidney disease (1.6%), and chronic respiratory diseases (1.2%). Last, the study sample included five pregnant women.

**Table 1:** Characteristics of the study population

Characteristic	*n*	COVID-19	Dengue	
		IgM+ or IgG+, % (95% CI)	IgG+, % (95% CI)	Active infection, % (95% CI)
Overall	326	78.0 (73.0–82.6)	88.0 (84.0–91.6)	13.0 (9.2–16.7)
District				
Belen	34	82.0 (65.0–93.2)	91.0 (76.0–98.1)*	15.0 (5.0–31.1)
Iquitos	107	73.0 (63.0–81.0)	92.0 (85.0–96.1)	16.0 (9.5–24.2)
Punchana	59	78.0 (64.0–88.0)	89.0 (77.0–95.8)	20.0 (11.0–33.5)
San Juan	126	82.0 (74.0–88.1)	84.0 (77.0–90.0)*	7.9 (3.9–14.1)
Gender				
Female	178	80.0 (73.0–85.4)	88.0 (83.0–92.5)	13.0 (8.8–19.4)
Male	148	76.0 (68.0–82.3)	88.0 (81.0–92.6)	11.0 (6.3–17.0)
Age, years				
18–29	88	81.0 (71.0–88.3)	84.0 (75.0–91.0)	13.0 (6.4–21.3)
30–59	196	77.0 (71.0–82.7)	89.0 (84.0–92.8)	12.0 (8.0–17.7)
> 60	42	86.0 (71.0–94.6)	88.0 (74.0–96.0)	9.5 (2.7–22.6)
Rural/urban				
Rural	39	87.0 (73.0–95.7)	86.0 (71.0–95.5)	5.1 (0.6–17.3)
Urban	287	77.0 (71.0–81.4)	89.0 (85.0–92.5)	14.0 (9.8–18.1)

* *P* < 0.05.

### Anti-DENV IgG seroprevalence estimate.

**Figure 2.**
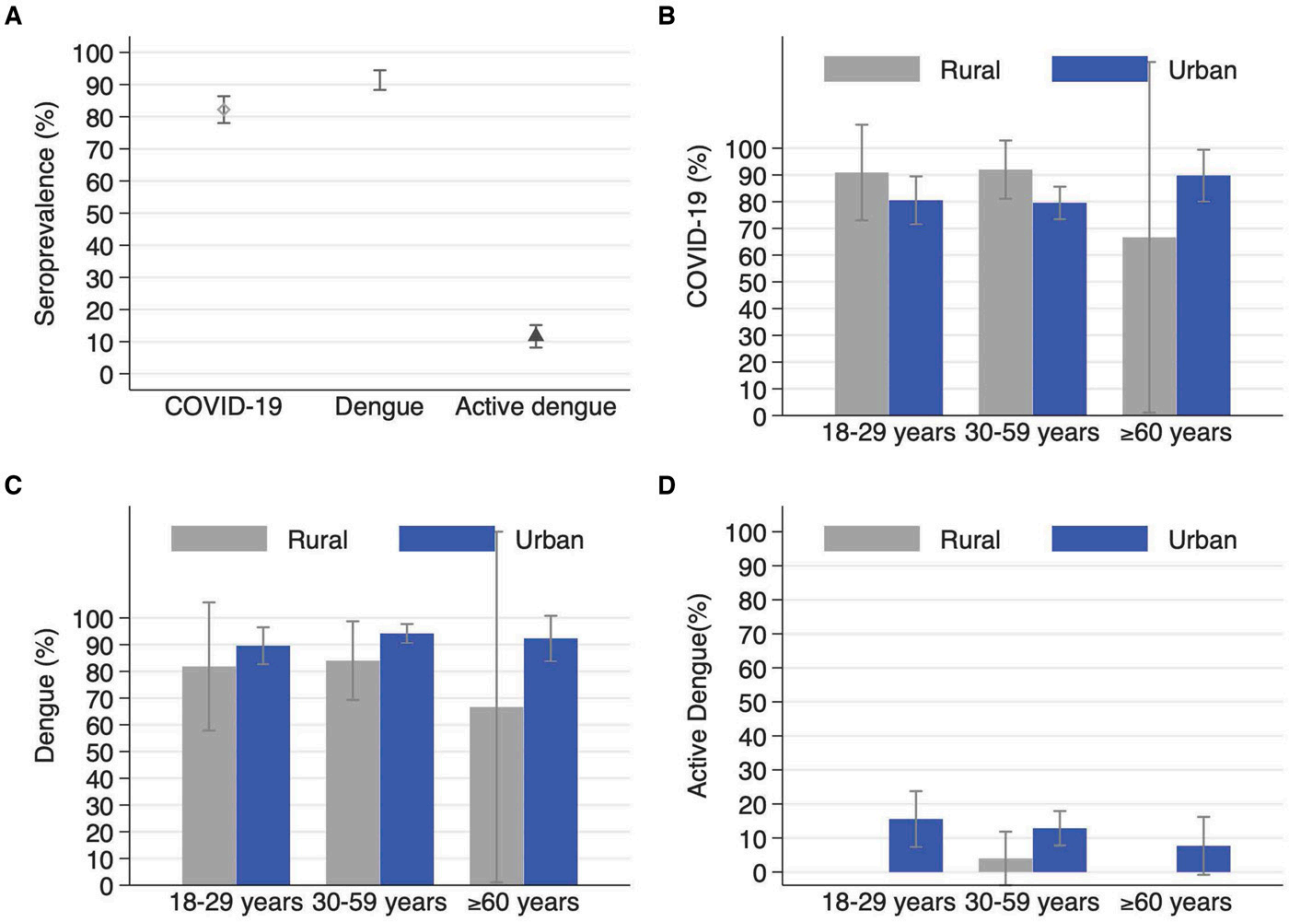
Seroprevalence of COVID-19, dengue, and active dengue among adults across Iquitos City overall (**A**) and disaggregated by age group and rural/urban area for COVID-19 seroprevalence (**B**) dengue seroprevalence (**C**), and dengue incidence (**D**).

**Figure 3.**
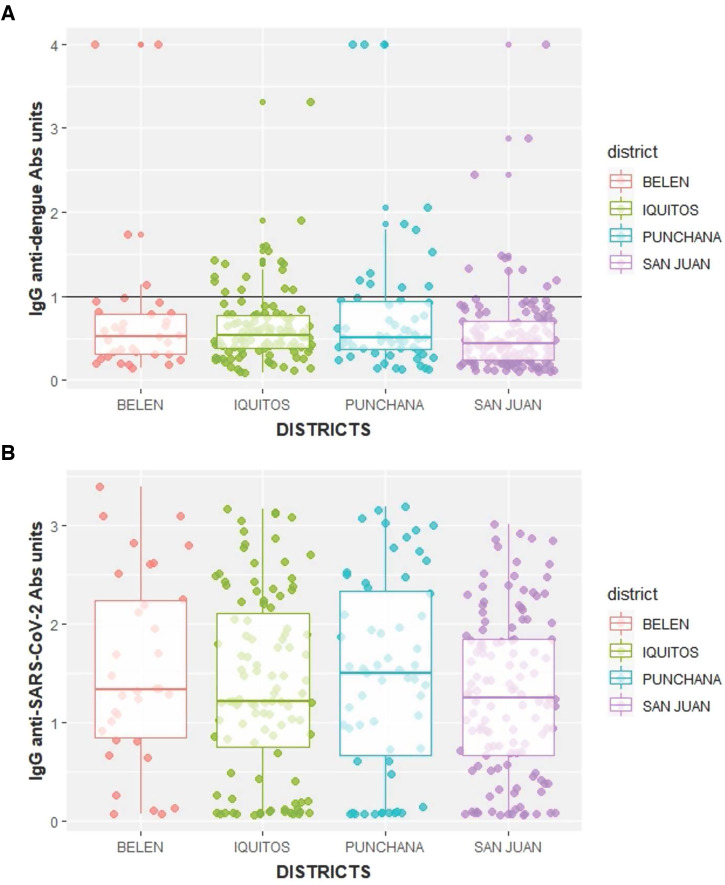
(**A**) Anti-dengue IgG antibody (Abs) absorbance units by Iquitos district. Active cases of dengue are located above the solid line. (**B**) COVID-19 IgG Abs absorbance units by Iquitos district.

We observed anti-DENV IgG positivity in 91.4% (298 of 326) of the adults, of which 81.3% also tested positive for anti-SARS-CoV-2 antibodies. After adjusting for the study sampling effects, and test sensitivity and specificity, we estimated an anti-DENV IgG seroprevalence of 88.0% (95% CI, 84.0–91.6). Anti-DENV IgG seroprevalence in Iquitos ranged from 84% (95% CI, 77–90) in the San Juan District to 92% (95% CI, 85.0–96.1) in the central Iquitos District. The only factor with a statistically significant prevalence ratio (PR) was the district, with San Juan having a lower prevalence compared with Belen (PR, 91.0; 95% CI, 76.0–98.1%). We did not observe significant differences by gender, age, and urban/rural areas (Figures 2 and 3A).

### Anti-SARS-CoV-2 antibody seroprevalence estimate.

We observed a seropositivity of 82.2% (268 of 326) either to IgM (4.3%) or IgG (81.3%) anti-SARS-CoV-2 antibodies. After adjusting for the study sampling effects, and test sensitivity and specificity, we estimated an anti-SARS-CoV-2 antibody seroprevalence of 78.0% (95% CI, 73.0–82.6) at baseline (Table 1). When disaggregating the anti-SARS-CoV-2 antibody seroprevalence by each specific antibody, we estimated a seroprevalence of anti-SARS-CoV-2 IgG of 78.0% (95% CI, 73.0–82.0) and a seroprevalence of anti-SARS-CoV-2 IgM of 3.6% (95% CI, 1.7–6.0). Overall, we observed that the anti-SARS-CoV-2 antibody seroprevalence in Iquitos did not vary significantly by age, gender, district, and urban/rural areas (Figures 2 and 3B).

### Association between the anti-SARS-CoV-2 and anti-DENV antibody seroprevalence.

We analyzed the association between our outcomes of interest and did not observe a significant association between anti-SARS-CoV-2 and anti-DENV antibody seroprevalence (PR, 0.96; 95% CI, 0.81–1.12; *P* = 0.576).

## DISCUSSION

Our study was conducted approximately 5 months after the peak of the first wave of COVID-19 transmission in the city of Iquitos, Peru. The anti-DENV antibody seroprevalence rates observed in this population-based sample were high, ranging from 84% to 92% (88% overall), consistent with seroprevalence rates reported for the city previously (neighborhood-specific age-adjusted DENV seroprevalence rates ranging from 67.1%–89.9% by plaque reduction neutralization test).^23^^,^^24^ Interestingly, in November 2019, cases caused by DENV-1 were first detected and increased until the March 14, 2020 quarantine declaration. Ministry of Health surveillance observed high numbers of DENV cases recorded during the 2019 to 2020 wet season, and most COVID-19 cases were reported during May 2020.^27^ After the declaration of the COVID-19 emergency, the reporting of DENV cases dropped dramatically in Latin America, most likely because of the impact of the pandemic on disease surveillance systems.^31^ Surveillance resources were diverted to the COVID-19 response, health-seeking behavior was strongly discouraged, and many febrile illnesses were attributed to COVID-19. In Iquitos, the health-care system eventually collapsed, resulting in no clear documentation of DENV case seroprevalence during that period. Our study attempted to gather data on the co-circulation of DENV that was likely occurring based on the observed increase of DENV-1 cases. This increase is significant because just before the quarantine declaration, DENV-1 replaced DENV-2 as the dominant serotype in the city and because there is an upsurge of DENV cases currently being observed by the recently reinitiated DENV surveillance (Dirección Regional de Salud Loreto, unpublished data).

The dengue virus did not disappear during the COVID-19 pandemic. On the contrary, the anti-DENV antibody seroprevalence remains high overall (> 84%). Although there was a statistical difference between the districts of San Juan and Belen, it is not easy to interpret these differences. More important is the documentation that seroprevalence in small surrounding communities was meager (12%) compared with urban sites in the city (88%). In many cases, this is because many of these communities have not been infested with the vector *Ae. aegypti*, but this is a very dynamic situation, with the distribution of this vector expanding with urbanization.^32^ In addition, this is the first report that compares anti-DENV antibody seroprevalence from the district of San Juan with the three northern districts of Iquitos City (Iquitos, Punchana, and Belen), which have been more extensively studied. In October 2009, during a vector control intervention trial, DENV seroprevalence was measured in the San Juan District for children and adults in 20 clusters of households, with an overall seroprevalence rate of 87% compared with 84% observed in our study.^33^ Previous studies indicate that transmission often begins in the central and northern districts (Iquitos and Punchana), and the southern districts are affect later (Belen and San Juan), which may explain why seroprevalence was slightly lower in the San Juan District in our study.

Another key finding of our study is that DENV and SARS-CoV-2 antibodies do not show cross-reactivity. Specifically, our results point in the direction that detecting antibodies IgG and IgM anti SARS-CoV-2 spike antibodies by ELISA in patients in patients with COVID-19 is not affected by prior exposure to DENV because the association among DENV and SARS-CoV-2 antibodies is insignificant or nonexistent. Such a finding has been described recently as supporting evidence of non-cross-reactivity between IgG anti-SARS-CoV-2 N Protein and IgG anti-DENV.^34^

Our results show an intense transmission for both diseases (DENV and COVID-19), which is not surprising considering the means of spread of these diseases and considering that Loreto region presents some socioeconomics that promotes such transmission: informal employment rates (81.8%), higher monetary poverty levels (28.3%–32.7%), and overcrowding.^28^ However, although the relationship between infectious diseases such as DENV and poverty is reported and known, more studies are needed to deepen and clarify the relationship between poverty and DENV and COVID-19 in the city of Iquitos.^35^ Our study describes the perfect scenario to assess the consequences of a DENV–COVID-19 co-circulation, combining the highest seroprevalence of COVID-19 observed in an adult cohort study worldwide and the already high seroprevalence of DENV reported in Iquitos City.

Our study was designed to estimate SARS-CoV-2 seroprevalence after the devastating first transmission wave. There was local knowledge that DENV cases were present, and the decision to amend this protocol to obtain some meaningful DENV data was made. Because of supply chain issues, the availability of DENV diagnostic kits was severely limited; for example, no IgM kits were available, leading to the selection of the kits used in our study. Because IgM levels in the early convalescent stage are significantly lower in secondary infections than in primary infections, the use of IgM tests may not have been useful in this context.^36^ In our study, we measured IgG anti-DENV levels that are generally detectable at low titers at the end of the first week of illness, increasing slowly after that, and that can be detected after several months and years.^37^ It is important to mention that it is likely that our estimated seroprevalence does not correspond entirely to DENV, but also includes a small fraction of anti-flavivirus antibodies caused by either Zika, yellow fever, and other flaviviruses as a result of the cross-reactivity of the technique, which is an important limitation in the determination of DENV seroprevalence in our study.

Another limitation was confining dengue testing to adults. Previous studies illustrate an increase of age-specific seroprevalence.^15^^,^^24^ Serotype-specific assays for neutralizing antibodies would also have been preferable, but was well beyond the scope of our project. If children had been tested and their seroprevalence for anti-DENV antibodies was as high or higher than observed in previous studies, this would have provided additional evidence of DENV circulation simultaneous to the COVID-19 pandemic. In this case, review board permission was not provided, illustrating the importance of establishing outbreak response protocols ahead of time when there is time to provide justification for sampling all age groups.

In conclusion, Iquitos City presented DENV and COVID-19 co-circulation, with one of the highest seroprevalences of anti-DENV and anti-SARS-CoV-2 antibodies worldwide. Furthermore, our study corroborates that DENV and COVID-19 have co-circulated with high transmission intensity in the Peruvian Amazon population, but with no correlation between their viral antibody levels. This evidence is highly relevant to similar settings because both diseases often require opposite recommendations. Although patients with DENV are encouraged to seek regular follow-up at their nearby health-care facility to prevent disease complications, COVID-19 patients with mild disease are encouraged to stay at home. To deal with this real conundrum, further studies are required to inform sound public health policies during co-circulation and syndemic situations.

## Financial Disclosure

This project was financed by DIRESA Iquitos and the Universidad Científica del Sur.

## Supplemental Materials


Supplemental materials

Supplemental materials
