# Supplementary figures and images for: Dengue and COVID-19 Co-Circulation in the Peruvian Amazon: A Population-Based Study
Source: Am J Trop Med Hyg. 2023 Apr 24;108(6):1249–55. doi: 10.4269/ajtmh.22-0539 (PMC10540116; doi:10.4269/ajtmh.22-0539)

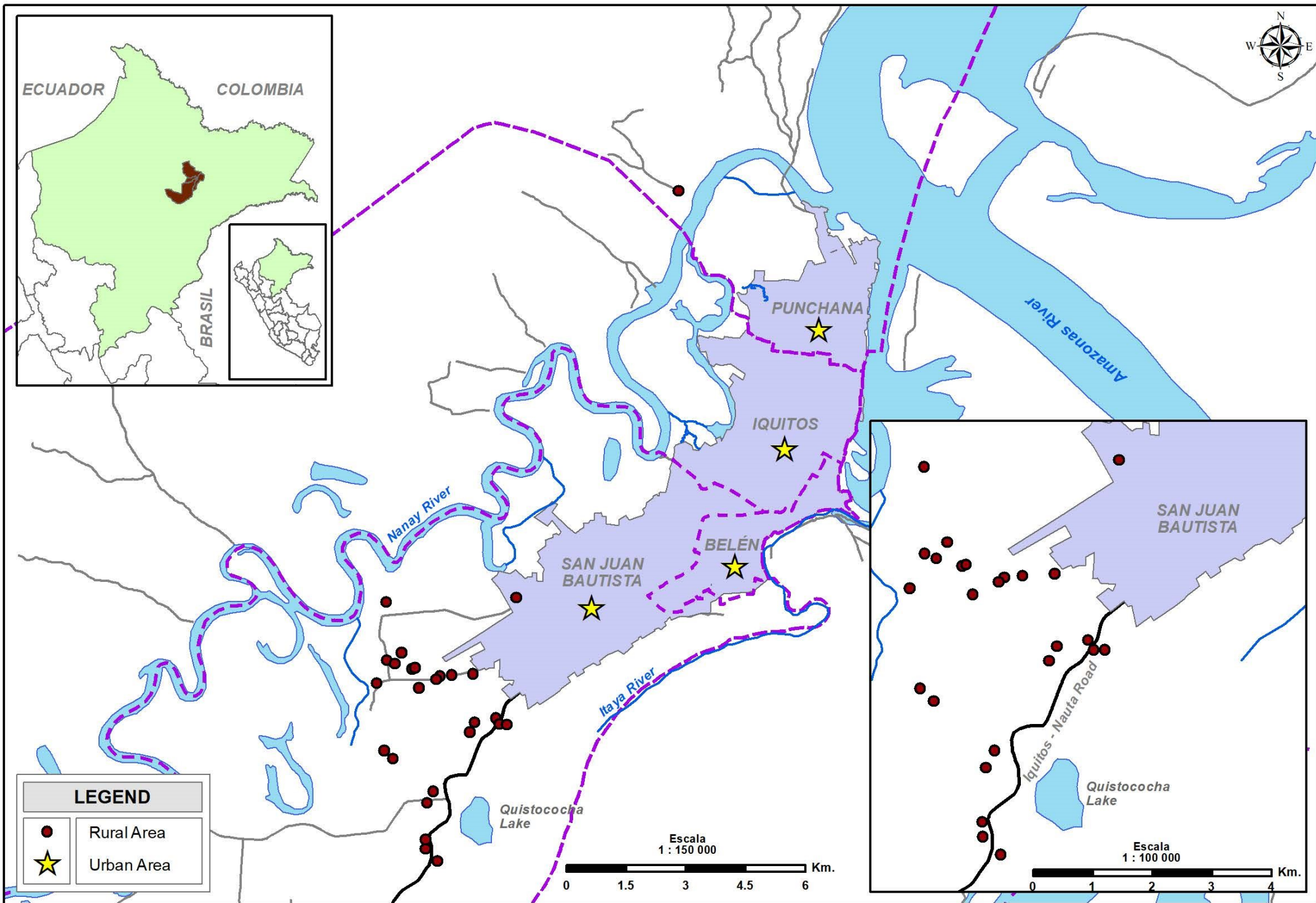

Supplement: Supplementary file 1 [file tpmd220539.SD1.pdf]
